# P-1363. Optimizing Management of Infections Caused by Metallo-β-Lactamase (MBL)–Producing Enterobacterales: An International Modified Delphi Study

**DOI:** 10.1093/ofid/ofaf695.1550

**Published:** 2026-01-11

**Authors:** Yehuda Carmeli, Mark A Price, George L Daikos, Marco Falcone, Ana C Gales, Stefen Hagel, Ali S Omrani, Paula Ramirez Galleymore, Subhash Todi, Minggui Wang, Maria Gheorghe, Brett Hauber, Christopher Little, Andrew Ian Townsend, Nathalie Baillon-Plot

**Affiliations:** Israel Ministry of Health, Tel Aviv, Tel Aviv, Israel; RTI Health Solutions, Pittsboro, North Carolina; National and Kapodistrian University of Athens, Athens, Attiki, Greece; Sapienza University of Rome, Rome, Lazio, Italy; UNIFESP, SAO PAULO, Sao Paulo, Brazil; Jena University Hospital, Jena, Thuringen, Germany; Qatar University College of Medicine, Hamad General Hospital, Doha, Al Wakrah, Qatar; La Fe University and Polytechnic Hospital, Valencia, Comunidad Valenciana, Spain; Manipal Hospital, Dhakuria, West Bengal, India; Institute of Antibiotics, Huashan Hospital, Fudan University, Shanghai, Shanghai, China (People's Republic); Pfizer Inc., Bucharest, Bucuresti, Romania; Pfizer, Inc., New York, New York; Pfizer Inc., Bucharest, Bucuresti, Romania; Pfizer Inc., Bucharest, Bucuresti, Romania; Pfizer Inc., Bucharest, Bucuresti, Romania

## Abstract

**Background:**

MBL-producing Enterobacterales pose an urgent global healthcare problem with limited treatment options. We aimed to gain a greater understanding of international perspectives on the management of serious infections due to MBL-producing Enterobacterales, using a modified Delphi process to assess expert-based opinion and establish consensus.

**Figure:**
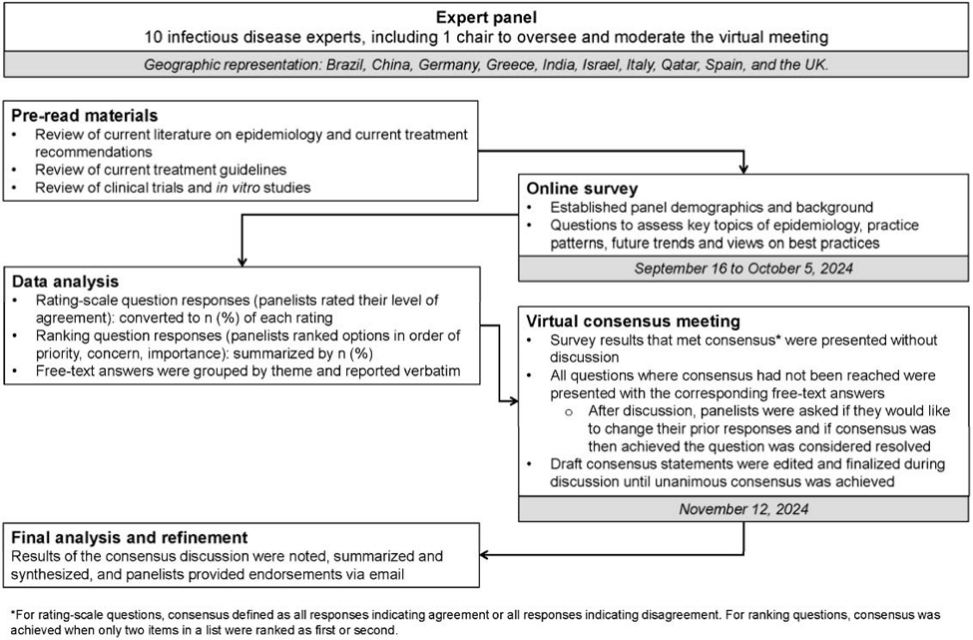

**Methods:**

Per Figure 1, a Delphi panel comprising 10 infectious disease experts with experience in treatment of antimicrobial-resistant bacterial infections completed a survey on epidemiology, disease, testing, and optimal management of infections caused by MBL-producing Enterobacterales. Draft consensus statements developed from the survey results were reviewed by the panel and either endorsed or edited to achieve unanimous consensus during a virtual meeting.

**Results:**

The consensus statements showed that all panelists identified MBL-producing Enterobacterales as a major source of antimicrobial resistance and infections in most countries. All panelists agreed that these infections are associated with high morbidity and mortality, potential need for critical-level care, increased length of hospitalization, and significant healthcare costs. In addition, the adverse impact of MBL was exacerbated by inappropriate use of multidrug antimicrobial regimens; hence, an MBL-targeting agent was deemed preferable. When aztreonam-avibactam (ATM-AVI) and cefiderocol are available, based on *in vitro* data showing activity of ATM-AVI against > 90% of New Delhi MBL-producing Enterobacterales, the preferred first-line treatment should be ATM-AVI. Where ATM-AVI and cefiderocol are not available, treatment options are limited to the co-administration of antimicrobials. The panel also highlighted key factors to guide earlier initiation of appropriate treatment: patient factors, local epidemiology, available testing, and drug characteristics.

**Conclusion:**

This Delphi panel achieved consensus on optimal management of MBL-producing Enterobacterales, finding preference for targeted MBL treatment, with monotherapy preferred where available.

Study sponsored by Pfizer Inc. A genAI tool (Pfizer) developed the 1st draft; authors assume content responsibility.

**Disclosures:**

Yehuda Carmeli, MD, Basilea: Advisor/Consultant|Enlivex: Advisor/Consultant|Omnix: Advisor/Consultant|Pfizer: Advisor/Consultant|Pfizer: Grant/Research Support|Roche: Advisor/Consultant Mark A. Price, MA, MEd, Pfizer: Advisor/Consultant George L. Daikos, MD, MSD: Honoraria|Pfizer: Honoraria|Pfizer: Travel expenses to attend ESCMID Global 2025|Roche: Advisor/Consultant Marco Falcone, MD, Gilead: Grant/Research Support|Infecto Pharm: Honoraria|Menarini: Honoraria|Pfizer: Honoraria|Shionogi: Honoraria|ViiV: Grant/Research Support Ana C. Gales, MD, BioMerieux: Advisor/Consultant|BioMerieux: Speaker|MSD: Advisor/Consultant|Pfizer: Advisor/Consultant|Pfizer: Speaker Stefen Hagel, MD, Gilead: Honoraria|Pfizer: Advisor/Consultant|Pfizer: Honoraria|Shionogi: Advisor/Consultant|Shionogi: Honoraria Ali S. Omrani, FRCP, FRCPath, BioMérieux: Advisor/Consultant|BioMérieux: Honoraria|Cepheid: Advisor/Consultant|Cepheid: Honoraria|Gilead: Advisor/Consultant|Gilead: Honoraria|GSK: Advisor/Consultant|GSK: Honoraria|Hikma: Advisor/Consultant|Hikma: Honoraria|MSD: Advisor/Consultant|MSD: Honoraria|Mundi Pharma: Advisor/Consultant|Mundi Pharma: Honoraria|Pfizer: Advisor/Consultant|Pfizer: Honoraria Maria Gheorghe, PhD, Pfizer: Stocks/Bonds (Public Company) Brett Hauber, PhD, Pfizer Inc: Stocks/Bonds (Private Company) Christopher Little, PhD, Pfizer: Stocks/Bonds (Public Company) Andrew Ian Townsend, PhD, Pfizer: Stocks/Bonds (Public Company) Nathalie Baillon-Plot, MD, Pfizer: Stocks/Bonds (Public Company)

